# Percutaneous retrieval of a biliary stent after migration and ileal perforation

**DOI:** 10.1186/1749-7922-4-6

**Published:** 2009-01-31

**Authors:** Derek M Culnan, Bryan J Cicuto, Harjit Singh, Robert A Cherry

**Affiliations:** 1 Department of Surgery, The Pennsylvania State University, College of Medicine, M. S. Hershey Medical Center, 500 University Drive, Hershey, PA 17033, USA; 2 Department of Radiology, The Pennsylvania State University, College of Medicine, M. S. Hershey Medical Center, 500 University Drive, Hershey, PA 17033, USA

## Abstract

We present a case of a migrated biliary stent that resulted in a distal small bowel perforation, abscess formation and high grade partial small bowel obstruction in a medically stable patient without signs of sepsis or diffuse peritonitis. We performed a percutaneous drainage of the abscess followed by percutaneous retrieval of the stent. The entero-peritoneal fistula closed spontaneously with a drain in place. We conclude, migrated biliary stents associated with perforation distal to the Ligament of Trietz (LOT), may be treated by percutaneous drainage of the abscess and retrieval of the stent from the peritoneal cavity, even when associated with a large intra-abdominal abscess.

## Case report

Endoscopic biliary stent placement is a well established, safe and minimally invasive modality for the treatment of biliary diseases such as choledocholithiasis.[1,2] Over the past decade the use of this modality has increased in prevalence and utility. Despite the overall safety of this modality, on rare occasions these stents may migrate from the biliary tract.[3] A small percentage of those stents perforate the gut and require surgical intervention.[4,5] We present an unusual case of biliary stent migration with distal small bowel perforation and abscess formation which was successfully treated using interventional radiology techniques, including percutaneous drainage and fluoroscopic removal of the stent.

A 76-year-old woman was admitted with cholecystitis and choledocholithiasis diagnosed via computed tomographic (CT) scan. Her past medical and surgical history was significant for paroxysmal atrial fibrillation, a right hemicolectomy and right oophorectomy for colon cancer, pulmonary embolism requiring inferior vena cava filter placement, endovascular abdominal aortic aneurysm repair, and a stroke resulting in vascular dementia. Endoscopic retrograde cholangiopancreatography (ERCP) with sphincterotomy was performed with removal of an impacted common bile duct stone and placement of an uncoated 10F plastic endostent, though the duct was radiographically clear. Four days later, after her liver function test normalized, she underwent a laparoscopic cholecystectomy during which an intra-peritoneal abscess was found surrounding a markedly inflamed and necrotic appearing gallbladder. The cholecystectomy was performed without complication and the abscess was drained adequately. The remainder of her post-operative course was unremarkable and she was discharged home on post-operative day five.

**Figure 1:**
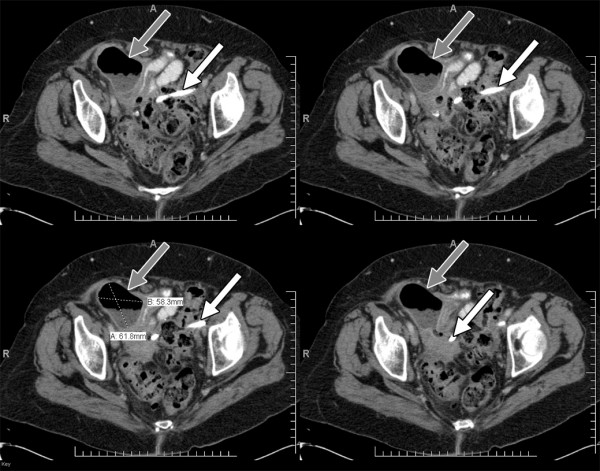
**CT Scan with right lower quadrant abscess**. Computer tomography images with intravenous and oral contrast demonstrating left lower quadrant abscess and small bowel obstruction. Grey arrows denote the abscess cavity. White arrows denote the endostent.

**Figure 2:**
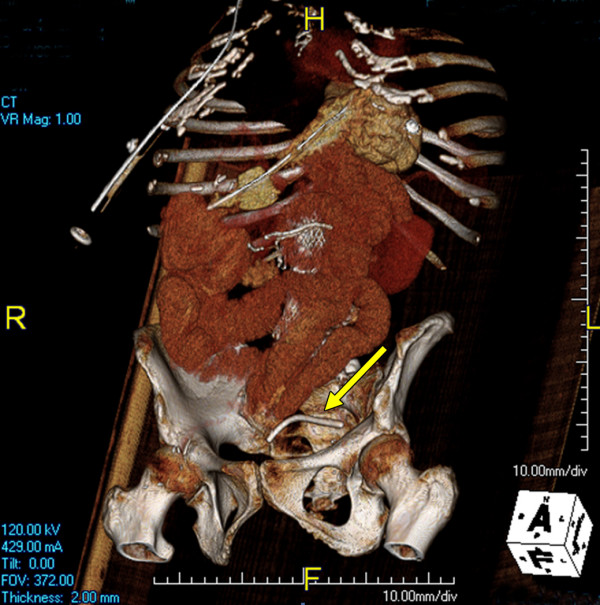
**CT Scan of the common bile duct stent**. 3-Dimensional reconstruction of CT data demonstrating the migrated biliary stent to be extraluminal in the left lower quadrant.

Approximately nine weeks after her laparoscopic cholecystectomy she presented to the emergency department complaining of four days of feculent emesis, intermittent diffuse abdominal pain, inability to tolerate *per os*, as well as obstipation for 24 hours. She denied any fevers or chills. An abdominal x-ray performed was consistent with a partial small bowel obstruction and a demonstrated a radiodense object consistent with a common bile duct stent overlying the lower pelvis. A CT scan was then performed which demonstrated a 5.8 × 6.2 cm abscess within the right lower quadrant with an extraluminal, radiodense biliary stent within the abscess cavity (Figure 1). Additionally there was no stent seen in the common bile duct. A three dimensional reconstruction of the CT scan confirmed that the common bile duct stent was extraluminal and in the left lower quadrant of the abdomen (Figure 2). A transition point of dilated small bowel was located adjacent to the abscess cavity. The patient missed her appointment to have the stent removed due to medical illness and was lost to follow-up by the endoscopist. Given her multiple comorbid conditions, hemodynamic stability, as well as the patient's strong desire to attempt non-operative management, the decision was made to immediately perform CT guided aspiration of the abscess with drain placement. This was possible because the patient had a localized abscess rather than diffuse peritonitis. Feculent-like material was aspirated without complication. The patient was also started on intravenous ciprofloxacin and metronidazole. She was followed with serial CT scans and abdominal examinations. Four days after the drainage procedure, the abscess cavity was noted to have decreased in size significantly. Her leukocytosis and bowel obstruction also resolved. However, six days after initial drainage, the abscess had subsequently increased in size and was associated with a decrease in drain output. Therefore the decision was made to upsize the drain.

**Figure 3:**
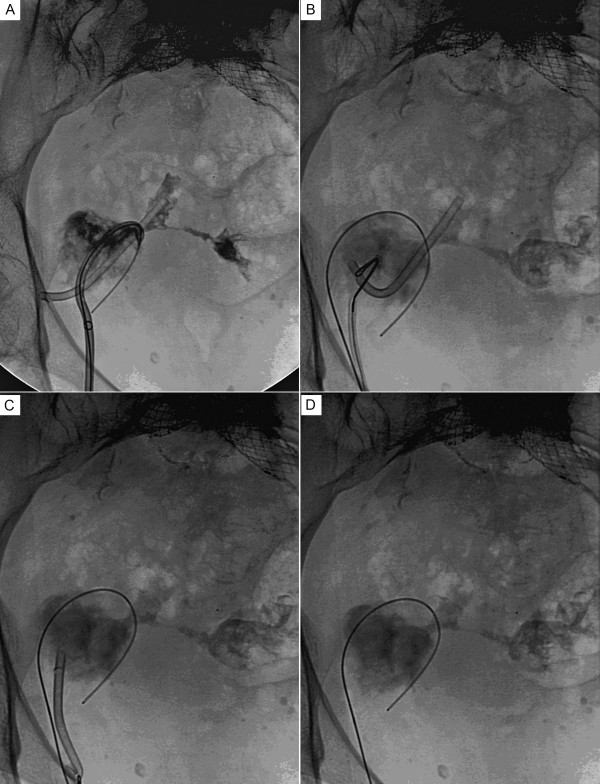
**Fluroscopic images of the extraluminal biliary stent**. Fluroscopic images demonstrating the retrieval of the extraluminal biliary stent. Panel A shows the catheter to be within the abscess cavity. Panel B shows the snare engaging the stent. Panel C shows the stent being removed through the sheath. Panel D shows the abscess cavity without the stent present.

Contrast was injected into the existing drain to confirm position then a guide wire was placed into the abscess via the drain (Figure 3A). The drainage catheter was replaced with a 7F sheath (Terumo Interventional Systems, Somerset, NJ) and a 25 mm Amplatz Gooseneck snare (EV3, Plymouth, MN) was advanced to capture the endostent (Figure 3B). The stent was then removed intact (Figure 3C, D) and a 12F multipurpose drain was placed. The stent was not able to be removed during the initial drainage because the collection had a teardrop configuration, with the drainage catheter at the top of the "tear" and the stent lying at the bottom of the collection. After percutaneous evacuation, the drainage catheter and the endostent came into proximity. At that point, removal was possible. A follow-up CT scan 2 days later demonstrated a decrease in the size of the abscess.

Her drainage continued at a stable and low level. She was discharged home with the drain with the intent of removing it after 6 weeks if there was no further an enteric or purulent content. Oral ciprofloxicin and metronidazole was prescribed three weeks.

**Figure 4:**
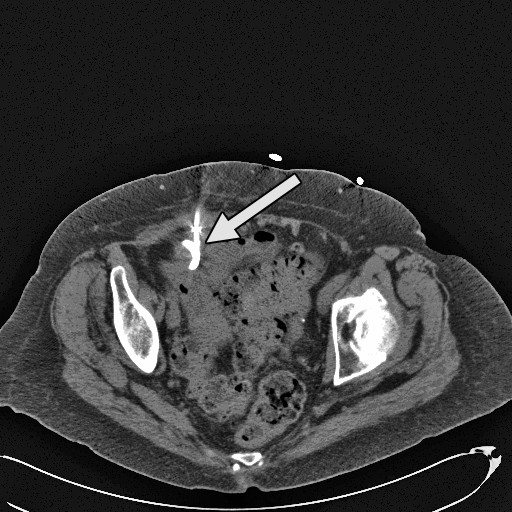
**CT image of collapsed abscess cavity**. CT image of the pelvis without contrast shows the drain in place and the abscess cavity completely collapsed.

During her outpatient visit three weeks later, she continued to drain about 10–20 cc per day of feculent material. A repeat abdominal and pelvic CT scan with contrast was performed (figure 4). The abscess had completely collapsed but a persistent fistulous connection was noted to the distal small bowel. The patient continued to do well clinically. We therefore decided to treat the patient conservatively as a controlled, low output enterocutaneous fistula by monitoring the drainage as an outpatient. Three weeks later, the character of the drainage changed to serosanguinous and the volume decreased to 2–3 cc per day. A repeat fluoroscopic contrast study of the drain showed resolution of the abscess and fistula. The drain was then removed without complication. Three months following drain removal, the patient was noted to be tolerating a regular diet with no signs of infection or fistula drainage. She suffered only mild deconditioning and had no significant loss of functional status.

## Discussion

Migration of endoscopically placed biliary stents is a well recognized complication of ERCP. Less than 1% of migrated stents cause intestinal perforation.[5] Of those that do perforate the bowel, the vast majority occur proximal to the ligament of Trietz (LOT). There have been a several case reports of intestinal perforation distal to the LOT, generally in the colon. [6-9] There have also been case reports describing small bowel perforation. [10-13]

Generally speaking, a double pigtail stent (7F) is preferable in cases involving choledocholithiasis. A straight stent may migrate since there is nothing to hold it in place, even though there are side flaps. An exception might be an impacted stone that is tight on the stent and prevents migration. Dislodged straight stents are more likely to perforate bowel whereas perforation with a pigtail is much more rare. Furthermore, straight 10 F plastic stents should generally be used for conditions such as strictures and tumours. The rationale for a double pigtail stent (7F) in this case is not known to the authors.

Migrated stents causing complications have either been retrieved endoscopically or via laparotomy.[4,7,14] There is at least one documented case of a percutaneous intervention to remove a biliary stent causing a retroperitoneal duodenal perforation and bilioma. However, there has not been a documented case involving percutaneous methods to retrieve a migrated stent beyond the LOT.

The existing literature on this subject would advocate prompt and aggressive surgical intervention because of gross contamination, intraperitoneal abscess, and bowel perforation.[4,5] Prompt surgical intervention is generally indicated for small bowel perforations, especially in the setting of a highly contaminated field, bowel obstruction and generalized abdominal pain. Historically, bowel perforation from migrated bilary stents has been treated either by endoscopic retrieval or laparotomy should endoscopic means fail. There are reports in which endoscopy is used to retrieve stents and close bowel perforations via clip application, but this only applies to areas that are accessible to endoscopic instrumentation.[14] In our case, endoscopic means was not possible because the perforation was in the distal small bowel and associated with a partial small bowel obstruction. Additionally, percutaneous interventions were undertaken in cases involving retroperitoneal bilomas.[15] Such bilomas were likely sterile, or at least not as heavily contaminated as an abscess.

Given the patient's past medical history, including advanced age, prior abdominal surgery, and cardiac status, we surmised that percutaneous drainage of the abscess posed a lower risk than a laparotomy. We concluded that drainage of the abscess would alleviate her small bowel obstruction, allow her inflammatory changes to resolve, and provide the time necessary for her to become nutritionally replete. In essence, we chose to treat this patient in a fashion similar to a complicated diverticular abscess or a perforated appendicitis with abscess formation. Prior reports involving biliary stent migration have advocated aggressive surgical intervention for patients with large infected intra-abdominal collections, delayed or critically ill clinical presentations, or a low physiologic reserve.[4,5] We had considered operative removal of the biliary stent after the patient had recovered clinically. However, the stent was able to be removed percutaneously during a drain upsizing. The patient had a 15 day hospital course and an extended period of percutaneous drainage. Of note, she initially refused operative intervention via laparoscopy or laparotomy to resect the enteroperitoneal fistula and preferred this treatment path.

## Conclusion

As percutaneous interventional techniques improve, cases that now require emergent surgical intervention may soon be better served by these less invasive techniques. In this circumstance, fluoroscopically guided percutaneous removal of a migrated biliary stent distal to the LOT, coupled with traditional conservative management principles in the treatment of enterocutaneous fistulas obviated the need for aggressive surgical intervention. This approach has not been previously documented. We conclude that fluoroscopic retrieval of migrated biliary stents associated with perforation distal to the LOT, along with percutaneous abscess drainage, may be a safe and effective treatment alternative to laparotomy for stable patients, even when associated with a large intra-abdominal abscess.

## Abbreviations

The following abbreviations were used in this manuscript: LOT: Ligament of Trietz; CT: Computed tomographic; ERCP: Endoscopic retrograde cholangiopancreatography.

## Competing interests

The authors declare that they have no competing interests.

## Authors' contributions

DMC drafted the manuscript. BJC, HS and RAC co-authored the writing of the manuscript. All authors participated in this case study. All authors read and approved the final manuscript.

## Consent

This activity was screened by our Institutional Review Board for exempt status according to the policies of this institution and the provisions of applicable regulations and was found not to require formal IRB review because it did not meet the regulatory definition of research.
